# Effectiveness of Collaborative Care for Depression and HbA1c in Patients with Depression and Diabetes: A Systematic Review and Meta-Analysis

**DOI:** 10.5334/ijic.6443

**Published:** 2022-08-30

**Authors:** Yanshang Wang, Mingzheng Hu, Dawei Zhu, Ruoxi Ding, Ping He

**Affiliations:** 1 School of Public Health, Peking University, 38 Xue Yuan Road, Haidian District, Beijing 100191, China; 2 China Center for Health Development Studies, Peking University, 38 Xue Yuan Road, Haidian District, Beijing 100191, China

**Keywords:** meta-analysis, diabetes, depressive symptoms, collaborative care

## Abstract

**Background and Aim:**

The collaborative care (CC) is emerging as an effective method in treating patients with multimorbidity, but evidence whether this model is effective for people with comorbid depression and diabetes is unclear. This study aimed to investigate whether CC could improve depression outcomes and HbA1c in patients with depressive symptoms and diabetes, and assess its effects on Quality of Life (QoL).

**Method:**

The author searched Embase, Scopus, PubMed, Cochrane, PsycINFO and CINAHL to identify randomized controlled trials (RCTs) and cluster RCTs published up to October 21, 2020. Studies were required to assess CC in patients with depressive symptoms and diabetes. The primary outcomes were depression treatment response rate and HbA1c and secondary outcome was Quality of Life (QoL). Available individual patient data was collected from all eligible studies. Studies were independently screened by two reviewers and critically appraised using the Cochrane Risk of Bias tool. This study conducted a systematic review and meta-analysis, and the fixed effects and random effects model were used to pool Relative Risks (RRs) and Standard Mean Differences (SMDs).

**Results:**

Our research identified 7906 articles, and finally 12 RCTs were included. Study sample sizes ranged from 58 to 417. The total follow-up period ranged from 12 weeks to 24 months. At follow-up, depression treatment response rate had a significant increase (RR = 1·31, 95% CI 1·23 to 1·39, I^2^ = 0%) in CC patients compared to controls. There was no statistically significant difference in HbA1c between CC group and the control group (SMD = 0·15, 95% CI -0·35 to 0·65, I^2^ = 97·6%). Overall QoL at follow-up was greater (SMD = 0·12, 95% CI 0·03 to 0·21, I^2^ = 54·2%) in CC patients compared to controls but the difference was minor.

**Conclusion:**

This systematic review and meta-analysis supported the effectiveness of CC in reducing depression and improving QoL in people with comorbid depression and diabetes.

## Introduction

There is growing awareness of the importance of multimorbidity, which is defined as patients living with two or more chronic health conditions [1]. Multimorbidity is a clinical challenge for patients and healthcare professionals, and a major challenge to healthcare systems that traditionally focused on acute care [2]. There may be no greater challenge than providing effective healthcare to patients living with mental and physical multimorbidity [3]. The health and social consequences of long-term comorbid mental and physical disorders are gradually regarded as key concerns for public health, as well as for economic and social development [4].

The multimorbidity of depression and diabetes can be recognized as a prototypical example of mental and physical multimorbidity [5]. Depressive symptom is common in people with type 2 diabetes (T2DM), with rates twofold higher than that in the general population [6], and it is linked to a significantly increased risk for diabetes [7]. Depression can reduce the control of HbA1c, blood pressure and cholesterol, treatment compliance, and overall health of patients with T2DM [8]. Besides, depression increases the subsequent risks of hyperglycemia, insulin resistance, and microvascular and macrovascular complications [9]. Moreover, the diagnosis of T2DM increases the risk of depression and can cause a more severe course of depression [9]. People with comorbid depression and diabetes are at higher risk of harm than people with only one of them or none [8].

The collaborative care (CC) model, as a typical type of integrated care, is emerging as an effective method in treating patients with multimorbidity. It can simultaneously reduce costs and improve clinical outcomes and treatment adherence in the management of mental and physical multimorbidity [10]. A systematic review has found that CC was associated with a significant improvement in depression outcomes and adherence in patients with depression and diabetes [11]. And a further systematic review with meta-analyses also found that the depression and glycaemia outcomes of the patients receiving CC were significantly improved [12].

However, existing systematic reviews have several limitations. First, participants were not just relevant to patients with depression and diabetes, some of which examined mixed groups of people with coronary artery disease, or other chronic illnesses. Therefore, these results may be biased and should be interpreted with caution. Second, these RCTs predominantly conducted in the USA, whether these results can be extrapolated to other countries and cultures worldwide remains unclear. Third, the current systematic reviews did not conduct subgroup analysis based on the duration of the intervention; that is, did not examine the short-term, mid-term, and long-term effects. Therefore, this systematic review makes several contributions to the existing literature on the impact of CC by addressing the above problems, which will assess the effectiveness of collaborative care on depression outcomes, HbA1c and QoL in patients with depressive symptoms and diabetes, and its heterogeneity in different periods.

## Method

This systematic review and meta-analysis were conducted in accordance with the Preferred Reporting Items for Systematic Reviews and Meta-analyses (PRISMA) Guidelines [13].

### Study Eligibility

Four criteria were taken into consideration: a. Only randomized controlled trials (RCTs) or cluster RCTs were eligible. b. Studies were required to have a baseline and at least one follow-up measure for depression and HbA1c. c. It is required to access to the full-text publication. d. There were no date restrictions.

#### Participants

Participants were eligible if they lived with diabetes and depression simultaneously.

1. Diagnosis of depression according to one of the followings: a. diagnosis made by primary care physician (PCPs); b. current prescription for antidepressant; c. diagnosis according to International Classification of Diseases (ICD-9 or ICD-10), Diagnostic and Statistical Manual (DSM-4 or DSM-5) and/or Research Diagnostic Criteria (RDC); d. assessment through clinician-rated and/or self-rated validated instruments; e. structured psychiatric interview [11].
2. Diagnosis of diabetes according to one of the followings: a. diagnosis made by PCP; b. current prescription for hypoglycemic agents; c. diagnosis according to ICD-9/ ICD-10; d. laboratory result [11].

#### Intervention

Primary care setting (community-based) was eligible.

Collaborative Care was defined as an integrated care model involving multidisciplinary healthcare providers, including: a. at least one health professional (eg, nurse, psychiatrist and/or psychologist) in addition to the PCP; b. a structured management plan that provides either pharmacological or nonpharmacological intervention for depression; c. scheduled patient follow-up and d. strengthened interprofessional communication between multidisciplinary team [14].

#### Comparators

Comparators was defined as either Usual Care (UC) or enhanced Usual Care (eUC). Usual Care (UC) was defined as standard care provided solely by PCP or nurses, and adding some simple contents on the basis of standard care was considered to be an enhanced service.

#### Outcomes

Primary outcomes: a. depression treatment response; b. HbA1c level. Secondary outcome: Quality of Life (QoL).

### Search Strategy and Identification of Studies

The search strategy was developed by the research team and checked by an information specialist prior to execution (appendix pp 1–14). One of the authors (YW) conducted the search of several databases: Embase, Scopus, PubMed, Cochrane, PsycINFO and CINAHL from inception to 27 October 2020. We reviewed reference lists of identified trials and systematic reviews to maximize the search for relevant articles.

### Study selection

After removing duplicates, two independent researchers (YW and MH) screened all titles and abstracts. After that, they obtained the full text of potentially eligible studies, and further screened them when the studies were deemed qualified or unclear. Disagreements over study eligibility finally reached consensus through discussion.

### Data collection process

Two independent researchers (YW and MH) extracted data by using a standardized, pre-piloted data extraction form. Information concerning the study characteristics, participants, intervention, comparators and outcomes were collected. The author contacted the author to obtain the data, when some studies mentioned outcomes of interest without providing estimates. Eight authors [15,16,17,18,19,20,21,22] were contacted for missing mean and standard deviations. And after 2 weeks, we had contacted those authors again. Finally, only one author provided this information [16].

### Assessment of risk of bias

Using the Cochrane Collaboration Risk of Bias tool, the quality of all included trials was assessed by two researchers (YW and MH) independently.

### Data Synthesis

The results of dichotomous variables were reported using the Relative Risks (RRs) with 95% Confidence Intervals (CIs) and Standard Mean Differences (SMDs) with 95% CI for continuous variables. Results were considered to be statistically significant if a *P* value less than 0.05. Moreover, the author assessed the heterogeneity of studies by observing the I^2^ value and the overlap of CIs on the forest plots [23]. The author used fixed effects models to pool outcomes if significant heterogeneity was not present (I^2^ < 50%); when significant heterogeneity was present, the author used random effects models (I^2^ ≥ 50%). Stata 15.0 was used for quantitative synthesis.

### Publication Bias

The funnel plot and Egger’s test were used to assess publication bias.

### Subgroup Analyses

The author conducted subgroup analyses based on length of follow-up (short-term: baseline to ≤3 months, medium-term: >3 months but ≤6 months, and long-term: >6 months).

### Sensitivity analyses

The author conducted sensitivity analyses by excluding trials with high or unknown risk of bias; excluding the largest trial; using random effect models. Besides, a sensitivity analysis was conducted by adding some studies that participants were DM2 and/or CHD patients with depressive symptoms, which had been included in previous reviews.

## Results

### Study Selection

**Figure 1:**
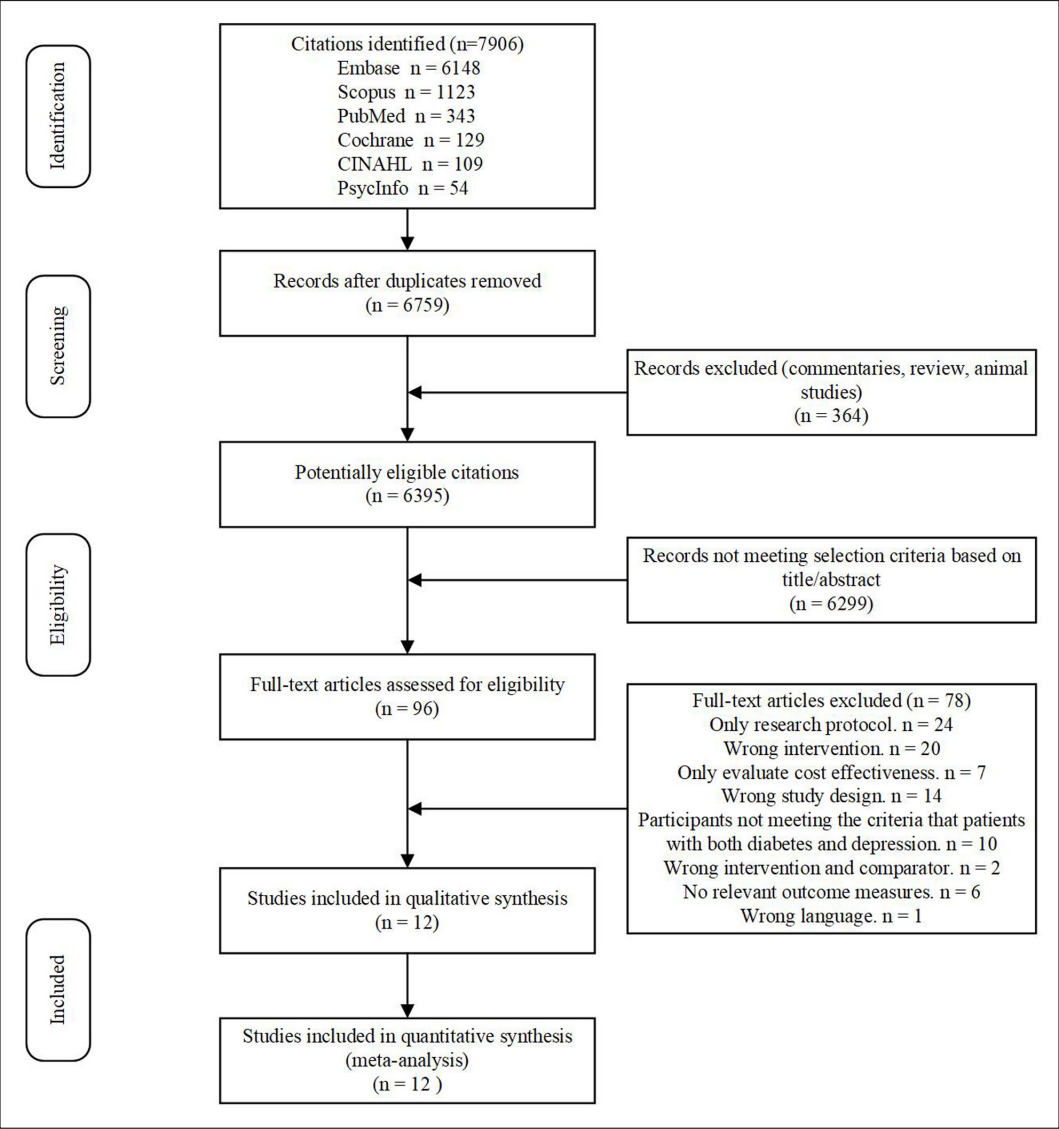
Flow Chart of Studies Selection.

This study identified 7906 articles (Figure 1). After removing duplicates (n = 1147), the titles and abstracts of 6759 papers were independently assessed for eligibility by two reviewers. Of these, 96 papers were deemed potentially relevant and assessed for eligibility in their full-texts; 84 articles were excluded. Finally, the study included 12 eligible studies in the meta-analysis [15,16,17,18,19,20,21,22,24,25,26,27].

### Characteristics of the Included Studies

Included studies were conducted in the US (n = 10) [15,17,19,20,21,22,24,25,26,27], Canada (n = 1) [16], and India (n = 1) [18] (Table 1).

**Table 1:** Characteristics of the Included Studies.


AUTHOR	YEAR	COUNTRY	INTERVENTION LENGTH	FEMALES IN TOTAL SAMPLE, N (%)	SAMPLE SIZE OF CC VS UC/EUC	METHOD OF DEPRESSION DIAGNOSIS	FOLLOW-UP	OUTCOME ASSESSMENT	CC=(baseline)		UC=(baseline)	
	
									MEAN DEPRESSION SCORE	MEAN HBA1C (%)	MEAN DEPRESSION SCORE	MEAN HBA1C (%)

Kathleen Ell	2005	USA	12-month	318(82.2)	193/194	PHQ-9≥10	6, 12, 18 month	SCL-20	1.7	9.01	1.41	9.05

de Vries McClintock	2014	USA	12-week	59(75.6)	37/41	Not clear	6, 12 week	CES-D	Not clear	7.5	Not clear	7.6

Ali	2015	India	12-month	239(59.2)	196/208	PHQ-9≥10	6, 12, 18, 24 mo	SCL-20	1.3	9.3	1.4	9

Naik	2012	USA	6-month	23 (10.2)	136/89	PHQ-9≥10	6, 12 mo	PHQ-9	15.8	9.2	16.2	9.3

Williams Jr	1999	USA	12-month	223(53.5)	205/212	structured clinical interview	6, 12 mo	SCL-20	1.67	7.3	1.72	7.3

Bogner	2010	USA	12-week	(102)56.7	92/88	a current prescription for an antidepressant	6, 12 week	PHQ-9	10.6	7.2	9.9	7

Bogner	2007	USA	12-week	49(84.5)	29/29	a current prescription for an antidepressant	2, 4, 6, 12 week	CES-D	15.6	7.3	19.7	7.3

Kathleen Ell	2005	USA	12-month	317(82)	193/194	PHQ-9≥10 and structured clinical interview	6, 12, 18, 24 month	SCL-20 and PHQ-9	Not clear	Not clear	Not clear	Not clear

Katon	2001	USA	12-month	214(65.1)	164/165	PHQ-9≥10 and SCL-20 ≥1.1	3, 6, 9, 12 month	SCL-90	1.7	8	1.6	8

Kathleen Ell	2013	USA	12-month	296(85)	178/170	PHQ-9≥10 and structured clinical interview	6, 12 month	SCL-20	1.01	8.91	1.06	8.84

Cummings	2017	USA	12-month	108(77.7)	67/72	DDS-2≥3 and/or PHQ-2 ≥ 3	6, 12 month	PHQ-12	9.7	9.88	8.8	9.35

Johnson	2010	Canada	12-month	87(55.4)	95/62	PHQ≥10	6, 12 month	PHQ-9	14.5	7.5	14.6	7.8


Abbreviations: CC, collaborative care; UC, Usual Care; eUC, enhanced Usual Care; PHQ-9, Patient Health Questionnaire–9; SCL-20, 20-item Symptom Checklist Depression Scale; CES-D, Center for Epidemiologic Studies Depression Scale; DDS-2, Diabetes Distress Scale-2; PHQ-2, Patient Health Questionnaire–2; PHQ-12, Patient Health Questionnaire–12; USA, United States.

#### Participants

Eight studies recruited participants with depressive symptoms using a validated questionnaire (PHQ-9, DDS-2, PHQ-2 and SCL-20) [15,16,18,19,22,27], three used a formal clinical diagnosis [20,24,25], two used a combination [21,26] and one was not clear [17]. Participants tended to be female (53.5%–85%) except one study (10.2%) [19]. Study sample sizes ranged from 58 to 417. The total follow-up period ranged from 12 weeks to 24 months. The period that the intervention group received CC ranged from 12 weeks to 12 months. There were 4 studies examined the sustained effect of the intervention [15,18,19,21].

#### Interventions

Interventions varied widely in terms of doses, communication methods (face-to-face or telephone), theory base (e.g., Problem-Solving Therapy (PST) or psychoeducation) and type of health care provider (e.g., nurses or social workers).

Interventions were heterogeneous and consisted of a multidisciplinary team (Case Manager (CM), psychiatrist and diabetologist) and a structured treatment plan (acute treatment and relapse prevention plan). In the 12 studies, only one has integrated a decision support electronic health record system into CC [18]. Interventions included processes to enhance interprofessional communication (caseload review meetings). The interventions were delivered partly within a stepped-care or shared-care framework and were presented to patients as a supplement, rather than a replacement.

#### Comparator

Seven comparators were UC [16,18,20,22,24,26,27], and 5 were eUC [15,17,19,21]. Enhanced Usual Care patients received standard clinic care and in addition were given health educational pamphlets [15,19,21], a care resource list [15,21] and individualized program to improve adherence [17].

### Outcomes

Three depression scales (PHQ-9, SCL-20 and CES-D) were used at various time-points (3–24 months) to measure depression scores (Table 1). Six studies reported the proportion of participants achieving depression treatment response and remission of depression at follow-up [15,18,19,21,22,26]; the change of depression scores at follow-up was calculated in 3 studies [16,17,20]. Three study reported the depression scores at follow-up Completely [24,27,28].

HbA1c could be obtained from 6 studies completely (Mean±SD) [15,21,24,25,26,27]; 3 studies recorded change of HbA1 [16,17,20] and one study showed the results with a line chart [22]. Only 2 studies published reduction in HbA1c for participants in this review [18,19]. Five studies provided the data of Qol [15,21,26].

Besides, through qualitative analysis, some studies reported other outcomes. For instance, care satisfaction (PACIC-11 and 5-point ordinal scale) was reported in 2 studies [16,22], and diabetes symptoms (Whitty-9) were reported in 2 studies [15,21]. The improved effects were found in CC group for diabetes symptoms, care satisfaction.

In these 12 studies, 7 studies used different methods to evaluate adherence to antidepressant regimens and oral hypoglycemic agents. In summary, these measurements could be divided into two categories: one was self-reported data [15,21,26,27] and the other was automatically monitored data [17,22,25,26]. A significant improvement in adherence to receiving care was seen in the CC group in comparison with the usual care group.

### Risk of Bias within Studies

All studies had a high RoB for personnel and participant blinding due to the nature of intervention. Of the 12 included studies, a high RoB was assigned to 3 for allocation concealment [16,17,20], 1 for blinding of outcome assessment [27], 1 for random sequence generation [16] and 2 for other bias [17,25] (appendix pp 15). An unclear RoB was assigned to 5 studies for incomplete outcome data [19,21,22,24,27], 3 for selective reporting [17,20,21], 2 for blinding of outcome assessment [15,17], 1 for allocation concealment [28] and 6 studies for other bias were unavailable [16,20,21,22,24,27].

### Effectiveness of Collaborative care for Depression

**Figure 2:**
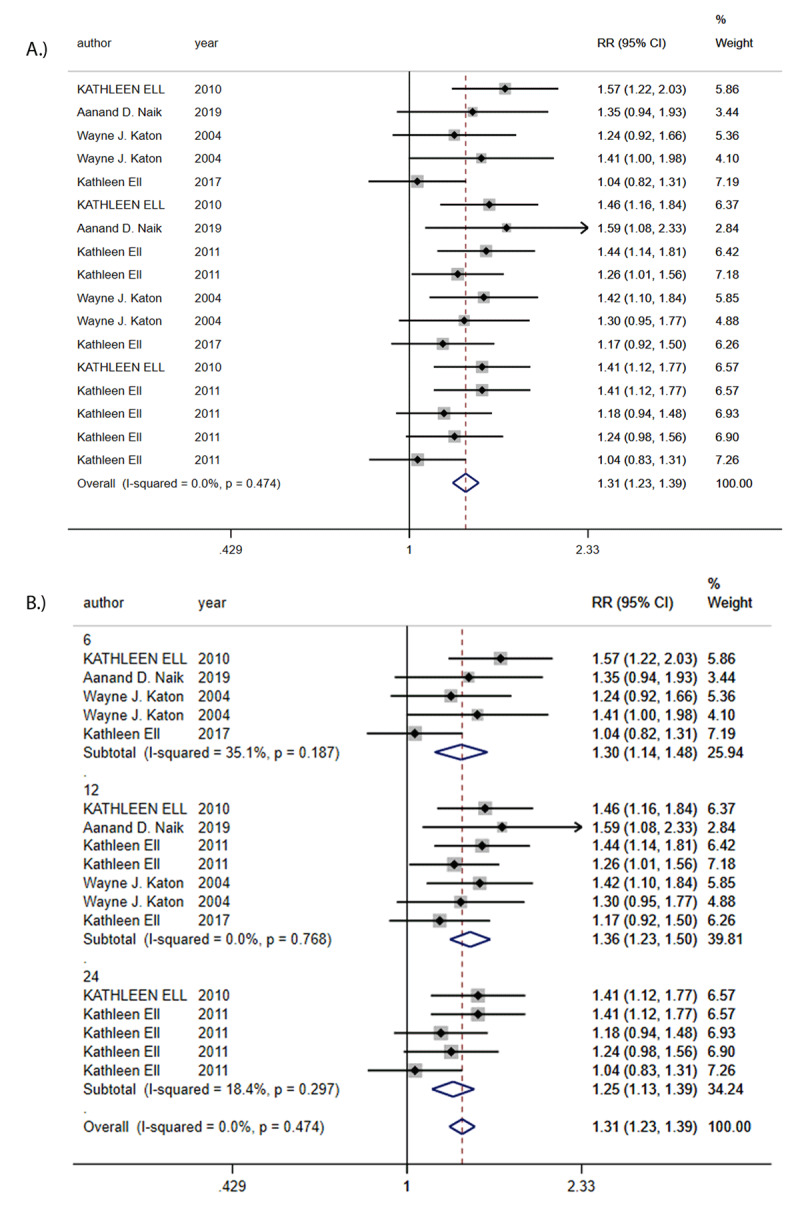
**A:** Risk Ratio (RR) in depression outcomes. **B:** Risk Ratios (RRs) of subgroup analysis in depression outcomes.

Six trials were used to calculate the overall effect size for depression outcomes. Collaborative Care was associated with significant increase in depression treatment response rate (RR = 1.31, 95% CI 1.23 to 1.39, I^2^ = 0%; Figure 2A). Funnel plot analysis showed no asymmetry; additionally, the Egger test (*P* = 0.259) and Begg test (*P* = 0.249) detected no significant small study effects. The meta-analysis results for treatment response rate were robust in sensitivity analyses. Subgroup analyses found that depression treatment response rate was significantly higher among trials with CC than in trials with usual care at short-term (RR = 1.30, 95% CI 1.14 to 1.48, I^2^ = 35.1%), medium-term (RR = 1.36, 95% CI 1.23 to 1.50, I^2^ = 0.00%) and long-term (RR = 1.25, 95% CI 1.13 to 1.39, I^2^ = 18.4%) (Figure 2B).

### Effectiveness of Collaborative Care for Diabetes

**Figure 3:**
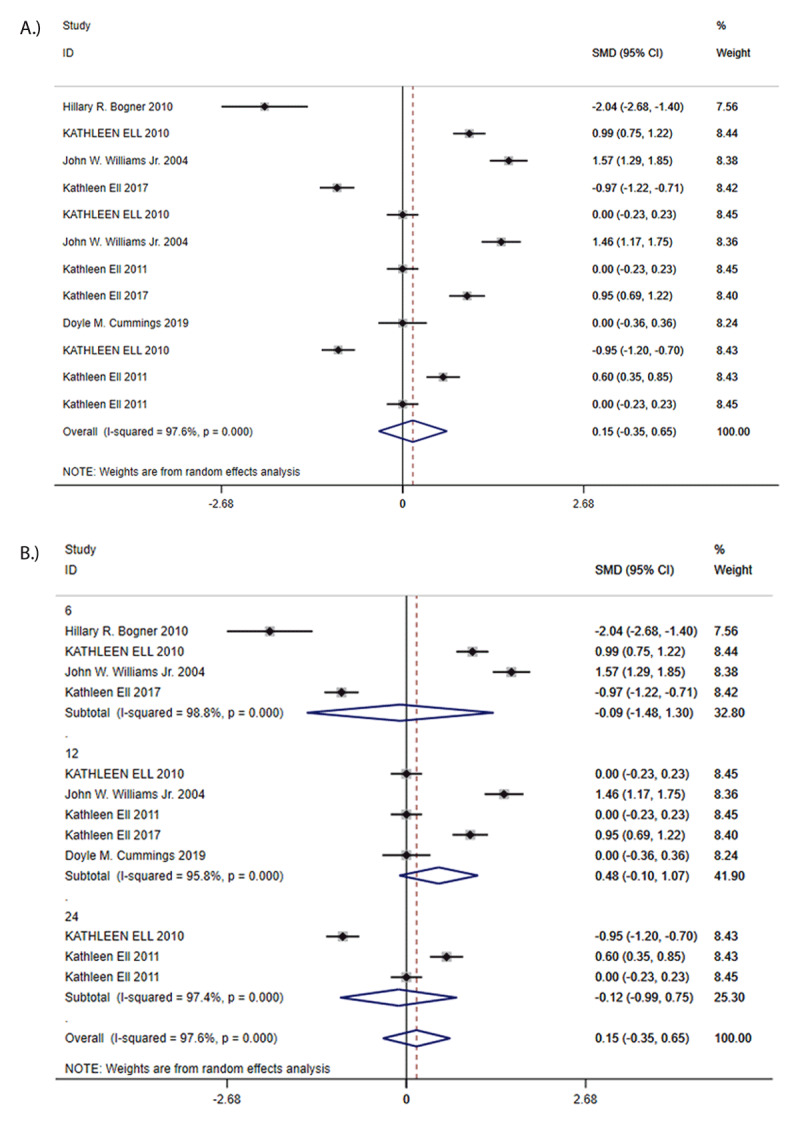
**A:** Standard Mean Differences (SMDs) in HbA1c. **B:** Standard Mean Differences (SMDs) of subgroup analysis in HbA1c.

Seven trials were used to calculate the overall effect size. There was no statistically significant difference in HbA1c between CC group and the control group (SMD = 0.15, 95% CI –0.35 to 0.65, I^2^ = 97.6%; Figure 3A). Funnel plot analysis showed no asymmetry; additionally, the Egger test (*P* = 0.641), and Begg test (*P* = 0.411) detected no significant small study effects. The meta-analysis results for treatment response rate were robust in sensitivity analyses. Subgroup analyses found that there was a non-significant reduction in HbA1c values in favor of CC at short-term (SMD = –0.09, 95% CI –1.48 to 1.30, I^2^ = 98.8%), medium-term (SMD = 0.48, 95% CI –0.10 to 1.07, I^2^ = 95.8%) and long-term (SMD = –0.12, 95% CI –0.99 to 0.75, I^2^ = 97.4%) (Figure 3B).

### Quality of Life

**Figure 4:**
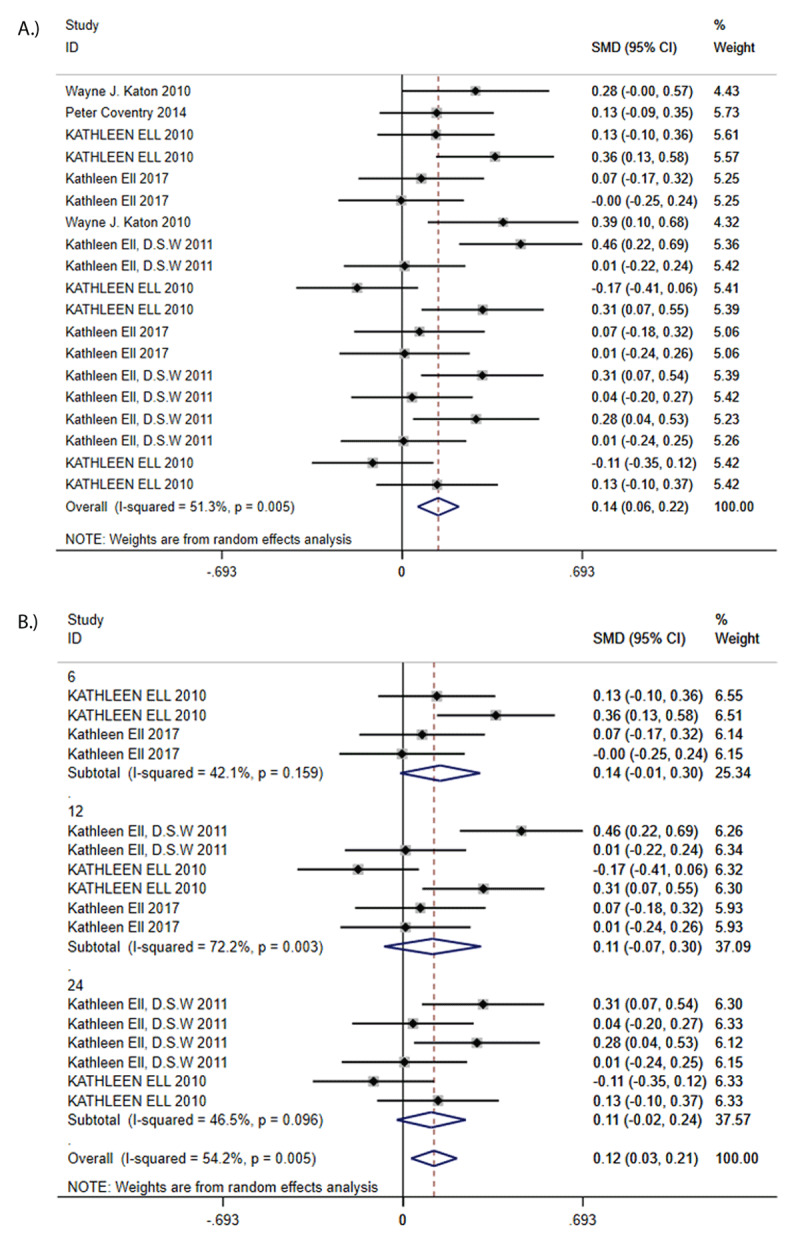
**A:** Standard Mean Difference (SMD) in QoL. **B:** Standard Mean Differences (SMDs) of subgroup analysis in Qol.

Overall QoL at follow-up was greater (SMD = 0.12, 95% CI 0.03 to 0.21, I^2^ = 54.2%; Figure 4A) in CC patients compared to controls but the difference was minor. Funnel plot analysis showed no asymmetry; additionally, the Egger test (*P* = 0.464), and Begg test (*P* = 0.964) detected no significant small study effects. The meta-analysis results for treatment response rate were robust in sensitivity analyses. Subgroup analyses found that there was no difference in QoL between CC and control group at short-term (SMD = 0.14, 95% CI –0.01 to 0.30, I^2^ = 42.1%), medium-term (SMD = 0.11, 95% CI –0.07 to 0.30, I^2^ = 72.2%) and long-term (SMD = 0.11, 95% CI –0.02 to 0.24, I^2^ = 46.5%) (Figure 4B). According to the size of the SMD, we can infer that the significance of the overall difference may come from short-term.

## Discussion

In the meta-analysis of 12 randomized controlled trials with a total of 3109 participants, CC significantly improved both depression outcome and Qol. However, there were no significant differences between CC and comparators patients regarding HbA1c.

### Principal Findings and Comparison with Other Studies

From the results, very limited information is available on the application and effectiveness of CC in Low- and Middle-Income Countries (LMICs). This study found that only one study from LMICs (India), US patients represent the largest proportion (82.0%). There is an urgent need for designing and implementing CC in the health systems of LMICs. The main point is probably the limited health resource in LMICs. Generally, whenever integrating CC into other contexts, especially in LMICs, economic development and cultural difference were two core dimensions that must be considered [29]. Under the situation of shortage of health resources in LMICs, it requires an innovative approach to existing health resources. In this study, there are some implications for scalability of CC from the India study, for instance, diabetes clinicians could be supported to manage depression [18].

All combined results showed CC is associated with significant improvements in depression outcomes. The results were consistent with the previous studies. Reductions in depression were greatest at mid-term follow-up, thus suggesting it took a period of time from the establishment of CC mode to the smooth operation. The results of on HbA1c was consistent with one previous review [11], but differ from the other [12]. A systematic review in 2014 by Evan Atlantis and colleagues that included 7 trials suggested a small effect on HbA1c level, but the methods used in this study is different from our study. Compared with these studies, the study excluded 6 trials with patients with comorbid other chronic illnesses. To determine whether our finding was driven by excluding trials, the author conducted one sensitivity analysis by adding trials that were originally excluded. When the author additionally included 6 RCTs, and the result was consistent and robust.

A systematic review in 2018 suggested multidisciplinary CC could significantly improve HbA1c [30]. However, it only included people with diabetes. Comparing with those without depression, diabetic patients with depression usually have more macrovascular and microvascular complications and worse glycemic control [29]. Besides, the prescription of antidepressant drugs, possible side-effects, and drug-drug interactions may cause bad glycemic control. Therefore, it is a complicated attempt to provide CC for patients with mental and physical multimorbidity for the existing health system, which is organized to deal with single illness and to separate mental and physical healthcare [3]. However, if each condition is considered in isolation, CC will not achieve the desired goals. CC needs to be carefully balanced against their possible benefits for mental and physical outcomes [29]. The future study should structure and standardize the process that integrate mental health into chronic care platforms by using the theoretical framework [28], rather than simply combine these two services. Through qualitative analysis, the study found that original research articles more often reported clinical outcomes at the patient level. However, there are few outcomes to measure patient’s real perception to CC. The measurement of these aspects played an important and indispensable role in the patient-centered care model. Besides, there are also few results on organizational factors at system level and factors of the healthcare providers, such as professional culture, financial management, communication patterns, implementation readiness, or presence of supportive leadership [4]. In order to reduce fragmented care and improve the continuity and coordination, the outcomes of CC in different levels should be considered Interventions varied widely in terms of doses, theory based and type of health care providers. No evidence exists to help elucidate independent contributions of each component and capture the mechanism, and no studies grouped and stratified according to the severity and persistence of conditions. It is not clear whether all the components of the CC are necessary, and what the mechanisms are. Only limited evidence exists about appropriate doses and treatment durations [29]. Besides, CC needed to develop and utilize the rapidly evolving and promising novel technologies, such as electronic health (eHealth) and mobile health (mHealth) applications, which could be integrated to the existing CC. In the 12 studies, only one incorporated a decision support electronic health record system into clinic workflows [18]. However, there is no intervention integrated provision of eHealth/mHealth technology for patients with depression and diabetes. To provide more precise and real people-centered care, it is necessary to consider the above problems.

### Limitations and Strengths

There are three limitations in our study. Firstly, some data have not been collected in this study, which suggests there were likely selective reporting here. Secondly, blinding participants to CC is challenging and may be problematic when outcomes are self-reported. The participants’ knowledge of their allocation could influence their responses and introduce social desirability bias [31]. However, when this study removed some studies with a high RoB, the primary results were not affected significantly. Thirdly, data meta-analysis based on individual participants remains vulnerable to important sources of bias. By detecting funnel plot asymmetry, publication bias is not likely to be present in the overall dataset.

This review has several strengths. First, a wide range of databases were searched, and the study followed the recommendations of the Cochrane Collaboration and PRISMA statement. Second, the study provided pure trial effects from the 12 trials that assessed people with depression and only diabetes, instead of reporting pooled estimates, and provide the latest evidence for CC.

## Conclusions

In conclusion, this review and meta-analysis supported the effectiveness of CC in reducing depression and improving QoL in people with comorbid depression and diabetes. It should be considered how to best incorporate them into the routine practice of LMICs and how to achieve the perfect integration of mental and physical care systems to maximize the improvements of both outcomes. Future research should focus on the effectiveness, feasibility and appropriateness of CC at different levels and aspects, and some new novel technologies should be considered in CC.

## Additional Files

The additional files for this article can be found as follows:

10.5334/ijic.6443.s1 Appendix 1. Search strategy.

10.5334/ijic.6443.s2 Appendix 2. Risk of Bias Assessment for the Included Studies.
