# Supplementary material for: Effectiveness of Collaborative Care for Depression and HbA1c in Patients with Depression and Diabetes: A Systematic Review and Meta-Analysis
Source: Int J Integr Care. 2022 Aug 30;22(3):12. doi: 10.5334/ijic.6443 (PMC9438466; doi:10.5334/ijic.6443)
Supplement: Appendix 1. — Search strategy. [file ijic-22-3-6443-s1.pdf]

Supplementary

Appendix 1: search strategy

PsycInfo:

#1 MA diabetes mellitus OR MA diabetes complications OR AB diabetes
OR (AB "IDDM" OR AB "NIDDM" OR AB "MODY" OR AB "T1DM" OR AB "T2DM" OR AB "T1D" OR AB "T2D")
NOT (MA diabetes insipidus OR ((AB diabetes OR AB insipidus)))（29921）

#2 MA depression OR MA depressive disorder
OR (AB depress* AND AB disorder*)
OR (AB depress* AND AB symptom*)
OR (AB depress* AND AB score*)
OR (AB depress* AND AB scale*)
OR (AB depress* AND AB unipolar)
OR ("affective disorder" OR AB "mood disorder")（256450）

#3 MA "patient care planning" OR TX "patient centered care" OR
OR MA Delivery of Health Care, Integrated OR MA comprehensive health care OR AB "comprehensive health care" OR AB "community based" OR MA Patient-Centered Care OR MA Patient Care Management
OR AB "patient management" OR MA Community Health Services OR MA Primary Health Care OR MA case management
OR AB "case management"
OR (AB "multidisciplinary care OR AB "multidisciplinary health" OR AB "multidisciplinary delivery" OR AB "multidisciplinary system" OR AB "multidisciplinary team")
OR (AB "collaborative care" OR AB "collaborative health" OR AB "collaborative delivery" OR AB "collaborative system" OR AB "collaborative team")
OR (AB "interdisciplinary care" OR AB "interdisciplinary health" OR AB "interdisciplinary delivery" OR AB "interdisciplinary system" OR AB "interdisciplinary team")
OR (AB "integrated care" OR AB "integrated health" OR AB "integrated delivery" OR AB "integrated system" OR AB "integrated team")
OR (AB "shared care" OR AB "shared health" OR AB "shared delivery OR AB "shared system" OR AB "shared team")
OR (AB "joint care" OR AB "joint health" OR AB "joint delivery OR AB "joint system" OR AB "joint team")（58137）

#4 (MA randomized controlled trials OR MA random allocation OR MA double-blind method OR MA single-blind method OR MA clinical trial
OR TX "clinical trial"
OR (AB "single blind" OR AB "double blind"　OR AB "triple blind")
OR TX random* OR TX placebo
OR TX "controlled trial" OR TX "controlled method" OR TX "controlled study"
OR TX "prospective trial" OR TX "prospective method" OR TX "prospective study"

OR TX crossover trial OR TX crossover design
OR TX cross-over trial OR TX cross-over design)
NOT (MA animal AND NOT (MA animal AND MA human))（304255）
#1 AND #2 AND #3 AND #4 (54)

CINAHL:
#1 MH diabetes mellitus OR MH diabetes complications OR AB diabetes
OR (AB "IDDM" OR AB "NIDDM" OR AB "MODY" OR AB "T1DM" OR AB "T2DM" OR AB "T1D" OR AB "T2D")
NOT (MH diabetes insipidus OR ((AB diabetes OR AB insipidus))) (169194)

#2 MH depression OR MH depressive disorder
OR (AB depress* AND AB disorder*)
OR (AB depress* AND AB symptom*)
OR (AB depress* AND AB score*)
OR (AB depress* AND AB scale*)
OR (AB depress* AND AB unipolar)
OR (AB "affective disorder" OR AB "mood disorder") (151076)

#3 TX "patient centered care" OR MH Delivery of Health Care, Integrated
OR MH comprehensive health care OR AB "comprehensive health care" OR AB "community based"
OR MH Patient-Centered Care OR MH Patient Care Management OR AB "patient management"
OR MH Community Health Services OR MH Primary Health Care OR AB case management OR AB "case management"
OR (AB "multidisciplinary care OR AB "multidisciplinary health" OR AB "multidisciplinary delivery" OR AB "multidisciplinary system" OR AB "multidisciplinary team")
OR (AB "collaborative care" OR AB "collaborative health" OR AB "collaborative delivery" OR AB "collaborative system" OR AB "collaborative team")
OR (AB "interdisciplinary care" OR AB "interdisciplinary health" OR AB "interdisciplinary delivery" OR AB "interdisciplinary system" OR AB "interdisciplinary team")
OR (AB "integrated care" OR AB "integrated health" OR AB "integrated delivery" OR AB "integrated system" OR AB "integrated team")
OR (AB "shared care" OR AB "shared health" OR AB "shared delivery OR AB "shared system" OR AB "shared team")
OR (AB "joint care" OR AB "joint health" OR AB "joint delivery OR AB "joint system" OR AB "joint team")（158178）

#4 (MH randomized controlled trials OR MH random allocation OR MH double-blind method OR MH single blind method OR MH clinical trial
OR TX "clinical trial"
OR AB "single blind" OR AB "double blind" OR AB "triple blind"
OR TX random* OR TX placebo
OR TX "controlled trial" OR TX "controlled method" OR TX "controlled study"
OR TX "prospective trial" OR TX "prospective method" OR TX "prospective study"
OR TX "crossover trial" OR TX "crossover design"
OR TX "cross-over trial" OR TX "cross-over design")
NOT (MH animal NOT (MH animal AND MH human)) (745216)
#1 AND #2 AND #3 AND #4 (109)

Cochrane:

#1 MeSH descriptor: [Diabetes Mellitus] explode all trees

#2 MeSH descriptor: [Diabetes Complications] explode all trees

#3 diabetes:ti,ab

#4 IDDM:ti,ab

#5 NIDDM:ti,ab

#6 MODY:ti,ab

#7 T1DM:ti,ab

#8 T2DM:ti,ab

#9 T1D:ti,ab

#10 T2D:ti,ab

#11 #1 OR #2 OR #3 OR #4 OR #5 OR #6 OR #7 OR #8 OR #9 OR #10

#12 MeSH descriptor: [Diabetes Insipidus] explode all trees

#13 diabetes:ab,ti AND insipidus:ti,ab

#14 #12 OR #13

#15 #11 NOT #14

#16 MeSH descriptor: [Depression] explode all trees

#17 MeSH descriptor: [Depressive Disorder] explode all trees

#18 (depress*) adj3 (disorder*):ti,ab

#19 (depress*) adj3 (symptom*):ti,ab

#20 (depress*) adj3 (score*):ti,ab

#21 (depress*) adj3 (scale*):ti,ab

#22 (depress* near/3 unipolar):ti,ab

#23 #18 OR #19 OR #20 OR #21 OR #22

#24 "affective disorder":ti,ab

#25 "mood disorder":ti,ab

#26 #16 OR #17 OR #23 OR #24 OR #25

#27 MeSH descriptor: [Patient Care Planning] explode all trees

#28 "patient centered care"

#29 "multidisciplinary care":ti,ab or "multidisciplinary health":ti,ab or "multidisciplinary delivery":ti,ab or "multidisciplinary system":ti,ab or "multidisciplinary team":ti,ab

#30 "interdisciplinary care":ti,ab or "interdisciplinary health":ti,ab or "interdisciplinary delivery":ti,ab or "interdisciplinary system":ti,ab or "interdisciplinary team":ti,ab

#31 "integrated care":ti,ab or "integrated health":ti,ab or "integrated delivery":ti,ab or "integrated system":ti,ab or "integrated team":ti,ab

#32 "collaborative care":ti,ab or "collaborative health":ti,ab or "collaborative delivery":ti,ab or "collaborative system":ti,ab or "collaborative team":ti,ab

#33 "shared care":ti,ab or "shared health":ti,ab or "shared delivery":ti,ab or "shared system":ti,ab or "shared team":ti,ab

#34 "joint care":ti,ab or "joint health":ti,ab or "joint delivery":ti,ab or "joint system":ti,ab or "joint team":ti,ab

#35 #29 OR #30 OR #31 OR #32 OR #33 OR #34

#36 MeSH descriptor: [Delivery of Health Care, Integrated] explode all trees

#37 MeSH descriptor: [Comprehensive Health Care] explode all trees

#38 "comprehensive health care":ti,ab
#39 "community based":ti,ab
#40 MeSH descriptor: [Patient-Centered Care] explode all trees
#41 MeSH descriptor: [Patient Care Management] explode all trees
#42 "patient management":ti,ab
#43 MeSH descriptor: [Community Health Services] explode all trees
#44 MeSH descriptor: [Primary Health Care] explode all trees
#45 MeSH descriptor: [Case Management] explode all trees
#46 "case management":ti,ab
#47 #27 OR #28 OR #35 #36 #37 OR #38 #39 OR #40 OR #41 OR #42 OR #43 OR #44 #45 OR #46
#48 (randomized controlled trial):pt
#49 MeSH descriptor: [Randomized Controlled Trial] explode all trees
#50 MeSH descriptor: [Random Allocation] explode all trees
#51 MeSH descriptor: [Double-Blind Method] explode all trees
#52 MeSH descriptor: [Single-Blind Method] explode all trees
#53 (clinical trial):pt
#54 MeSH descriptor: [Clinical Trial] explode all trees
#55 "clinical trial".ti,ab
#56 "single blind":ti,ab or "double blind":ti,ab or "triple blind":ti,ab
#57 random*
#58 placebo
#59 "controlled trial" or "controlled method" or "controlled study"
#60 "prospective trial" or "prospective method" or "prospective study"
#61 "crossover trial" or "crossover design"
#62 "cross-over trial" or "cross-over design"
#63 #48 OR #49 OR #50 OR #51 OR #52 OR #53 OR #54 OR #55 OR #56 OR #57 OR #58 OR #59 OR #60 OR #61 OR #62
#64 #15 AND #26 AND #47 AND #63 (129)

PubMed:

#1 "diabetes mellitus"[MeSH Terms]

#2 "diabetes complications"[MeSH Terms]

#3 "diabetes"[Title/Abstract]

#4 "IDDM"[Title/Abstract] OR "NIDDM"[Title/Abstract] OR "MODY"[Title/Abstract] OR "T1DM"[Title/Abstract] OR "T2DM"[Title/Abstract] OR "T1D"[Title/Abstract] OR "T2D"[Title/Abstract]

#5 #1 OR #2 OR #3 OR #4

#6 "diabetes insipidus"[MeSH Terms]

#7 "diabetes"[Title/Abstract] AND "insipidus"[Title/Abstract]

#8 #6 OR #7

#9 #5 NOT #8

#10 "depression"[MeSH Terms]

#11 "depressive disorder"[MeSH Terms]

#12 "depress*"[Title/Abstract] AND "disorder*"[Title/Abstract]

#13 "depress*"[Title/Abstract] AND "symptom*"[Title/Abstract]

#14 "depress*"[Title/Abstract] AND "score*"[Title/Abstract]

#15 "depress*"[Title/Abstract] AND "scale*"[Title/Abstract]

#16 "depress*"[Title/Abstract] AND "unipolar"[Title/Abstract]

#17 #12 OR #13 OR #14 OR #15 OR #16

#18 affective disorder*[Title/Abstract]

#19 mood disorder*[Title/Abstract]

#20 #10 OR #11 OR #17 OR #18 OR #19

#21 "patient care planning"[MeSH Terms]

#22 "patient centered care"[Text Word]

#23 "delivery of health care, integrated"[MeSH Terms]

#24 "comprehensive health care"[MeSH Terms]

#25 "comprehensive health care"[Title/Abstract]

#26 "community based"[Title/Abstract]

#27 "patient-centered care"[MeSH Terms]

#28 "patient care management"[MeSH Terms]

#29 "patient management"[Title/Abstract]

#30 "community health services"[MeSH Terms]

#31 "primary health care"[MeSH Terms]

#32 "case management"[MeSH Terms]

#33 "case management"[Title/Abstract]

#34 collaborative care[Title/Abstract] OR collaborative health[Title/Abstract] OR collaborative delivery [Title/Abstract] OR collaborative system[Title/Abstract] OR collaborative team[Title/Abstract]

#35 integrated care[Title/Abstract] OR integrated health[Title/Abstract] OR integrated delivery [Title/Abstract] OR integrated system[Title/Abstract] OR integrated team[Title/Abstract]

#36 interdisciplinary care[Title/Abstract] OR interdisciplinary health[Title/Abstract] OR interdisciplinary delivery [Title/Abstract] OR interdisciplinary system[Title/Abstract] OR interdisciplinary team[Title/Abstract]

#37 multidisciplinary care[Title/Abstract] OR multidisciplinary health[Title/Abstract] OR multidisciplinary delivery [Title/Abstract] OR multidisciplinary system[Title/Abstract] OR multidisciplinary team[Title/Abstract]

#38 shared care[Title/Abstract] OR shared health[Title/Abstract] OR shared delivery [Title/Abstract] OR shared system[Title/Abstract] OR shared team[Title/Abstract]

#39 joint care[Title/Abstract] OR joint health[Title/Abstract] OR joint delivery [Title/Abstract] OR joint system[Title/Abstract] OR joint team[Title/Abstract]

#40 #21 OR #22 OR #23 OR #24 OR #25 OR #26 OR #27 OR #28 OR #29 OR #30 OR #31 OR #32 OR #33 OR #34 OR #35 OR #36 OR #37 OR #38 OR #39

#41 "randomized controlled trial"[Publication Type]

#42 "random allocation"[MeSH Terms]

#43 "double-blind method"[MeSH Terms]

#44 "single-blind method"[MeSH Terms]

#45 "clinical trial"[Publication Type]

#46 "clinical trial"[Text Word]

#47 single blind[Title/Abstract] OR double blind[Title/Abstract] OR triple blind[Title/Abstract]

#48 "random*"[Text Word]

#59 "placebo"[Text Word]

#50 controlled trial[Text Word] OR controlled method[Text Word] OR controlled study[Text Word]

#51 prospective trial[Text Word] OR prospective method[Text Word] OR prospective study[Text Word]

#52 crossover trial[Text Word] OR crossover design[Text Word]

#53 cross-over trial[Text Word] OR cross-over design[Text Word]

#54 #41 OR #42 OR #43 OR #44 OR #45 OR #46 OR #47 OR #48 OR #49 OR #50 OR #51 OR #52 OR #53

#55 animal[MeSH Terms]

#56 human[MeSH Terms]

#57 #55 AND #56

#58 #55 NOT #57

#59 #54 NOT #58

#61 #9 AND #20 AND #40 AND #59（343）

Scopus:

#1 (INDEXTERMS ("diabetes mellitus") OR INDEXTERMS ("diabetes complications") OR TITLE-ABS (diabetes)
OR TITLE-ABS ("IDDM") OR TITLE-ABS ("NIDDM") OR TITLE-ABS ("MODY") OR TITLE-ABS ("T1DM") OR TITLE-ABS ("T2DM") OR TITLE-ABS ("T1D") OR TITLE-ABS ("T2D"))
AND NOT (INDEXTERMS ("diabetes insipidus") OR (TITLE-ABS (diabetes) AND TITLE-ABS ("insipidus"))) （881789）

#2 INDEXTERMS(depression) OR INDEXTERMS("depressive disorder")
OR (TITLE-ABS(depress*) PRE/3 (TITLE-ABS(disorder*) OR TITLE-ABS("symptom*") OR TITLE-ABS("score*") OR TITLE-ABS("scale*")))
OR (TITLE-ABS("unipolar") W/3 TITLE-ABS("depressi*"))
OR (TITLE-ABS("affective disorder*") OR TITLE-ABS("mood disorder*")) （564418）

#3 ("patient care planning") OR ALL("patient centered care")OR INDEXTERMS("delivery of health care, integrated") OR INDEXTERMS("comprehensive health care")
OR TITLE-ABS("comprehensive health care") OR TITLE-ABS("community based") OR INDEXTERMS("patient-centered care") OR INDEXTERMS("patient care management")
OR TITLE-ABS("patient management") OR INDEXTERMS("community health services") OR INDEXTERMS("primary health care") OR INDEXTERMS("case management") OR TITLE-ABS("case manegement")
OR TITLE-ABS("collaborative care") OR TITLE-ABS("collaborative health") OR TITLE-ABS("collaborative delivery") OR TITLE-ABS("collaborative system") OR TITLE-ABS("collaborative team")
OR TITLE-ABS("integrated care") OR TITLE-ABS("integrated health") OR TITLE-ABS("integrated delivery") OR TITLE-ABS("integrated system") OR TITLE-ABS("integrated team")
OR TITLE-ABS("interdisciplinary care") OR TITLE-ABS("interdisciplinary health") OR TITLE-ABS("interdisciplinary delivery") OR TITLE-ABS("interdisciplinary system") OR TITLE-ABS("interdisciplinary team")
OR TITLE-ABS("multidisciplinary care") OR TITLE-ABS("multidisciplinary health") OR TITLE-ABS("multidisciplinary delivery") OR TITLE-ABS("multidisciplinary system") OR TITLE-ABS("multidisciplinary team")
OR TITLE-ABS("shared care") OR TITLE-ABS("shared health") OR TITLE-ABS("shared delivery") OR TITLE-ABS("shared system") OR TITLE-ABS("shared team")
OR TITLE-ABS("joint care") OR TITLE-ABS("joint health") OR TITLE-ABS("joint delivery") OR TITLE-ABS("joint system") OR TITLE-ABS("joint team")（406261）

#4 ((INDEXTERMS("randomized controlled trial") OR INDEXTERMS("random allocation") OR INDEXTERMS("double-blind method")
OR INDEXTERMS("single-blind method") OR INDEXTERMS("clinical trial") OR ALL("clinical trial") OR TITLE-ABS("single blind")
OR TITLE-ABS("double blind") OR TITLE-ABS("triple blind") OR ALL("random*") OR

ALL("placebo") OR ALL("controlled trial")
OR ALL("controlled method") OR ALL("controlled study") OR ALL("prospective trial") OR ALL("prospective method")
OR ALL("prospective study") OR ALL("crossover trial") OR ALL("crossover design") OR ALL("cross-over trial")
OR ALL("cross-over design")) AND NOT (INDEXTERMS(animal) AND NOT (INDEXTERMS(animal) AND INDEXTERMS(human))))（11363558)
#1 AND #2 AND #3 AND #4 (1124)

Embase:

#61.	#9 AND #20 AND #40 AND #60	6,148	21 Oct 2020
#60.	#55 NOT #59	7,703,912	21 Oct 2020
#59.	#56 NOT #58	5,506,668	21 Oct 2020
#58.	#56 AND #57	22,530,588	21 Oct 2020
#57.	'human'/exp	22,530,588	21 Oct 2020
#56.	'animal'/exp	28,037,256	21 Oct 2020
#55.	#41 OR #42 OR #43 OR #44 OR #45 OR #46 OR #47 OR #48 OR #49 OR #50 OR #51 OR #52 OR #53 OR #54	9,764,352	21 Oct 2020
#54.	'cross-over trial' OR 'cross-over design'	10,785	21 Oct 2020
#53.	'crossover trial' OR 'crossover design'	22,456	21 Oct 2020
#52.	'prospective trial' OR 'prospective method' OR 'prospective study'	702,528	21 Oct 2020
#51.	'controlled trial':de OR 'controlled method':de OR 'controlled study':de	7,971,587	21 Oct 2020
#50.	'placebo'	467,543	21 Oct 2020
#49.	'random*'	1,806,461	21 Oct 2020
#48.	'single blind':ab,ti OR 'double blind':ab,ti OR 'triple blind':ab,ti	215,769	21 Oct 2020
#47.	'clinical trial'/exp	1,540,416	21 Oct 2020
#46.	'clinical trial'	1,592,625	21 Oct 2020
#45.	'single blind method'/exp	40,381	21 Oct 2020
#44.	'double blind method'/exp	177,021	21 Oct 2020
#43.	'random allocation'/exp	88,267	21 Oct 2020
#42.	'randomized controlled trial'/exp	625,462	21 Oct 2020
#41.	'randomized controlled trial'	825,630	21 Oct 2020
#40.	#21 OR #22 OR #23 OR #24 OR #25 OR #26 OR #27 OR #28 OR #29 OR #30 OR #31 OR #32 OR #33 OR #34 OR #35 OR #36 OR #37 OR #38 OR #39	5,484,329	21 Oct 2020
#39.	'joint care':ab,ti OR 'joint health':ab,ti OR 'joint delivery':ab,ti OR 'joint system':ab,ti OR 'joint team':ab,ti	1,663	21 Oct 2020
#38.	'shared care':ab,ti OR 'shared health':ab,ti OR 'shared delivery':ab,ti OR 'shared system':ab,ti OR 'shared team':ab,ti	2,396	21 Oct 2020
#37.	'multidisciplinary care':ab,ti OR 'multidisciplinary health':ab,ti OR 'multidisciplinary delivery':ab,ti OR 'multidisciplinary system':ab,ti OR 'multidisciplinary team':ab,ti	34,038	21 Oct 2020
#36.	'interdisciplinary care':ab,ti OR 'interdisciplinary health':ab,ti OR 'interdisciplinary delivery':ab,ti OR 'interdisciplinary system':ab,ti OR	7,586	21 Oct 2020

	'interdisciplinary team':ab,ti		
#35.	'integrated care':ab,ti OR 'integrated health':ab,ti OR 'integrated delivery':ab,ti OR 'integrated system':ab,ti OR 'integrated team':ab,ti	15,508	21 Oct 2020
#34.	'collaborative care':ab,ti OR 'collaborative health':ab,ti OR 'collaborative delivery':ab,ti OR 'collaborative system':ab,ti OR 'collaborative team':ab,ti	4,159	21 Oct 2020
#33.	'case management':ab,ti	13,640	21 Oct 2020
#32.	'case management'/exp	11,870	21 Oct 2020
#31.	'primary health care'/exp	172,109	21 Oct 2020
#30.	'community health services'/exp	126,585	21 Oct 2020
#29.	'patient management':ab,ti	28,812	21 Oct 2020
#28.	'patient care management'/exp	841,548	21 Oct 2020
#27.	'patient-centered care'/exp	841,548	21 Oct 2020
#26.	'community based':ab,ti	76,553	21 Oct 2020
#25.	'comprehensive health care':ab,ti	1,038	21 Oct 2020
#24.	'comprehensive health care'/exp	5,411,539	21 Oct 2020
#23.	'delivery of health care, integrated'/exp	11,357	21 Oct 2020
#22.	'patient centered care'	7,170	21 Oct 2020
#21.	'patient care planning'/exp	30,265	21 Oct 2020
#20.	#10 OR #11 OR #17 OR #18 OR #19	559,724	21 Oct 2020
#19.	'mood disorder':ab,ti	8,082	21 Oct 2020
#18.	'affective disorder':ab,ti	9,255	21 Oct 2020
#17.	#12 OR #13 OR #14 OR #15 OR #16	219,264	21 Oct 2020
#16.	(depress* NEAR/3 unipolar):ab,ti	6,458	21 Oct 2020
#15.	(depress* NEAR/3 scale*):ab,ti	66,139	21 Oct 2020
#14.	(depress* NEAR/3 score*):ab,ti	27,859	21 Oct 2020
#13.	(depress* NEAR/3 symptom*):ab,ti	103,708	21 Oct 2020
#12.	(depress* NEAR/3 disorder*):ab,ti	78,796	21 Oct 2020
#11.	'depressive disorder'/exp	507,733	21 Oct 2020
#10.	'depression'/exp	507,733	21 Oct 2020
#9.	#5 NOT #8	1,132,703	21 Oct 2020
#8.	#6 OR #7	16,759	21 Oct 2020
#7.	'diabetes':ab,ti AND 'insipidus':ab,ti	11,241	21 Oct 2020
#6.	'diabetes insipidus'/exp	15,303	21 Oct 2020
#5.	#1 OR #2 OR #3 OR #4	1,145,139	21 Oct 2020
#4.	iddm:ab,ti OR niddm:ab,ti OR mody:ab,ti OR t1dm:ab,ti OR t2dm:ab,ti OR t1d:ab,ti OR t2d:ab,ti	93,618	21 Oct 2020
#3.	diabetes:ab,ti	809,994	21 Oct 2020
#2.	'diabetes complications'/exp	152,553	21 Oct 2020
#1.	'diabetes mellitus'/exp	1,015,811	21 Oct 2020
