# Supplementary material for: Effectiveness of Collaborative Care for Depression and HbA1c in Patients with Depression and Diabetes: A Systematic Review and Meta-Analysis
Source: Int J Integr Care. 2022 Aug 30;22(3):12. doi: 10.5334/ijic.6443 (PMC9438466; doi:10.5334/ijic.6443)
Supplement: Appendix 2. — Risk of Bias Assessment for the Included Studies. [file ijic-22-3-6443-s2.pdf]

Appendix 2: Risk of Bias Assessment for the Included Studies

Studies	Year	Selection bias		Performance bias	Detection bias	Attrition bias	Reporting bias	Other bias
		Random sequence generation	Allocation concealment	Blinding of participants and personnel	Blinding of outcome assessment	Incomplete outcome data	Selective reporting	Anything else, ideally prespecified
Kathleen Ell	2005	+	+	-	?	+	+	+
de Vries McClintock	2014	+	-	-	?	+	?	-
Ali	2015	+	+	-	+	+	+	+
Naik	2012	+	+	-	+	?	+	+
Williams Jr	1999	+	+	-	+	?	+	?
Bogner	2010	+	-	-	+	+	?	?
Bogner	2007	+	?	-	+	+	+	-
Kathleen Ell	2005	+	+	-	+	?	?	?
Katon	2001	+	+	-	+	?	+	?
Kathleen Ell	2013	+	+	-	+	+	+	+
Cummings	2017	+	+	-	-	?	+	?
Johnson	2010	-	-	-	+	+	+	?

Notes: - high risk of bias; + low risk of bias; ? unclear risk of bias.
